# Iatrogenic intrapericardial diaphragmatic hernia diagnosed by cardiovascular magnetic resonance

**DOI:** 10.1186/1532-429X-12-3

**Published:** 2010-01-08

**Authors:** Brandon C Drafts, Haroon L Chughtai, Daniel W Entrikin

**Affiliations:** 1 Department of Internal Medicine, Wake Forest University School of Medicine, Winston-Salem, NC 27157, USA; 2 Department of Cardiology, Saint Joseph Mercy Oakland Hospital, Pontiac, MI 48341, USA; 3 Departments of Radiology and Internal Medicine Section on Cardiology, Wake Forest University School of Medicine, Winston-Salem, NC 27157, USA

## Abstract

Intrapericardial diaphragmatic hernias are very uncommon and are most typically caused by high-force blunt trauma. Other iatrogenic causes such as prior surgical formation of a pericardial window have been described, but are exceedingly rare. We present a case of an intrapericardial diaphragmatic hernia in a patient with a prior pericardial window in which the diagnosis was unclear using conventional imaging modalities, but was established using cardiovascular magnetic resonance.

## Background

Intrapericardial diaphragmatic hernias are very uncommon, and although they may be congenital they are more typically the consequence of prior trauma, interventional procedures, or prior surgery. If these pericardial hernias result in the presence of bowel within the pericardial sac, visualization of the heart and pericardial contents with echocardiography can be difficult or impossible. The limitation of echocardiography in this diagnosis is related to the fact that ultrasound beams are not transmitted through gas-containing structures such as bowel. Newer advanced multiplanar imaging modalities such as cardiovascular magnetic resonance (CMR) are not as affected by the presence of gas-containing structures around the heart, and thus allow for comprehensive visualization of the heart and pericardial contents to establish the correct diagnosis.

## Case Presentation

**Figure 1:**
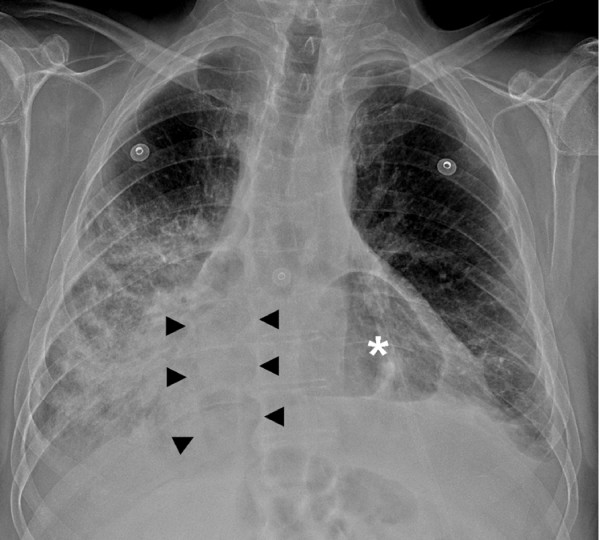
**Chest Radiograph**. Chest radiograph demonstrating the stomach bubble (white asterisk) projecting over the heart and a portion of the transverse colon that communicates with the abdominal cavity superimposed over the right heart border (black arrowheads).

**Figure 2:**
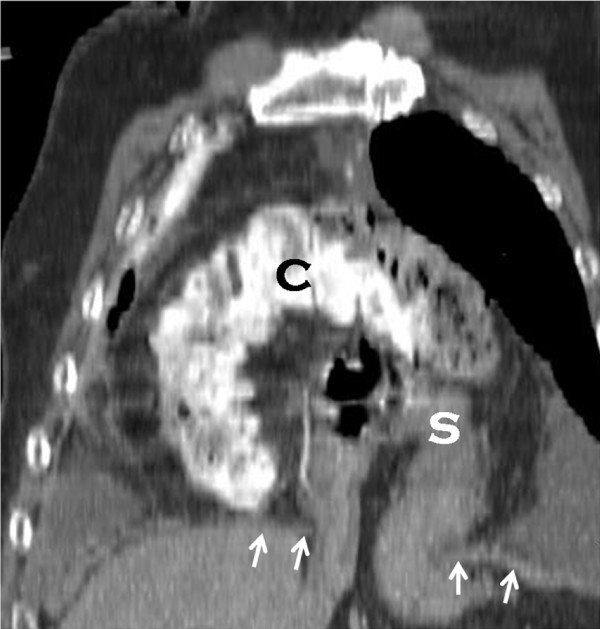
**Chest Computed Tomography Scan**. Sagittal oblique multiplanar reformat (MPR) from outside CT scan demonstrating discontinuity of diaphragm (white arrows) through which portions of the stomach (S) and transverse colon (C) have herniated.

A 70 year-old Caucasian male presented to our institution as a transfer for further evaluation and management of his presenting symptoms of dyspnea, chest pain, and a sensation of chest fullness. The patient had a complicated medical history including recurrent bilateral pleural effusions, treated by multiple prior thoracenteses that failed to provide long-term symptomatic relief. Furthermore, the patient also had recurrent pericardial effusions that required a pericardial window three years prior to his presentation to our institution. At the time of hospital admission, the patient was hemodynamically stable and his physical exam was only remarkable for pulsus paradoxus and bibasilar rhonchi. Electrocardiogram (ECG) showed a normal sinus rhythm and normal axis, but had low voltage throughout. Chest radiograph (figure 1) demonstrated portions of the stomach and colon projecting over the heart. A transthoracic echocardiogram was then ordered for further assessment, but the heart could not be visualized. Review of a recent thoracic computed tomographic (CT) scan (figure 2) demonstrated transdiaphragmatic herniation of portions of the stomach and transverse colon. However, because the CT scan was a routine nongated examination, image quality was not sufficient to differentiate between intrapericardial herniation versus intramediastinal herniation. CMR was requested for further evaluation.

**Figure 3:**
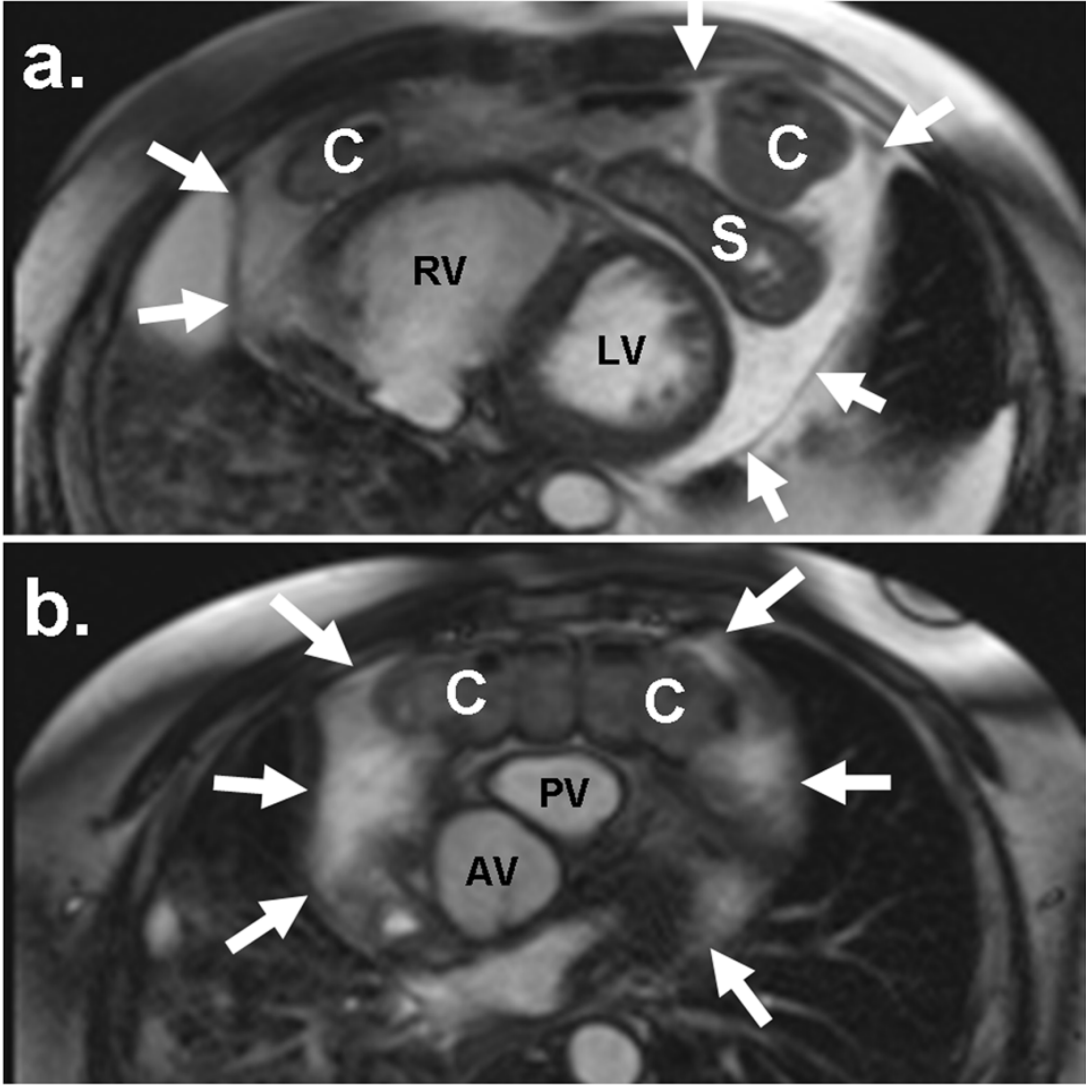
**Cardiovascular Magnetic Resonance**. (a) Axial true FISP image through the lower pericardial sac demonstrating portions of the stomach (S) and transverse colon (C) within the pericardial sac (white arrows), surrounding the right (RV) and left (LV) ventricles. (b) Axial true FISP image at the level of the aortic (AV) and pulmonic (PV) valves demonstrating a long segment of transverse colon within the pericardial sac.

CMR revealed a segment of the transverse colon and portions of the stomach within the pericardial sac anterior to both ventricles (figure 3; Additional Files 1 &2). This finding confirmed a diagnosis of an intrapericardial diaphragmatic hernia. The nonobstructive bowel communicated with the peritoneal cavity thru a defect in the inferior pericardium that measured 6.5 × 5.4 cm. Cardiac function was preserved with a left ventricular ejection fraction of 64% and normal left ventricular wall motion. The patient remained in stable condition throughout his hospital course and his presenting symptoms improved with adjustments in his medical therapy. He was evaluated by the cardiothoracic surgery team and was determined to be high risk for surgical intervention given his other co-morbid medical problems. He was discharged home and has been followed as an outpatient for continued management.

## Discussion

Intrapericardial diaphragmatic hernias have three known etiologies: traumatic, congenital, or iatrogenic. Traumatic herniation is the most common cause and is usually due to high-force blunt injuries or penetrating traumas to the chest or abdomen [1]. Congenital causes are secondary to a developmental failure of the septum transversum, which can create a pericardio-peritoneal opening [2]. Iatrogenic causes are extremely rare and can be caused by prior coronary artery bypass surgery [3], insertion of a pacemaker via an abdominal approach [4], or, as in the case we present, subxiphoid pericardial window for recurrent pericardial effusions.

Clinical presentations are variable and commonly include nonspecific cardiopulmonary or gastrointestinal symptoms that might delay the diagnosis. Prior reports have shown more critical presentations with associated complications including visceral strangulation, ischemic bowel, or cardiac tamponade, which would indicate emergent surgical reduction and repair [5]. Our patient presented with pulsus paradoxus on physical exam with low voltage on ECG that might suggest cardiac tamponade in an unstable patient. However, our patient remained hemodynamically stable throughout his hospital course with preserved cardiac function. He also denied any abdominal pain, nausea, vomiting, or constipation that would suggest gastrointestinal compromise.

In the case we present, the diagnosis was established with CMR. The initial chest radiograph demonstrated a potential mediastinal hernia with the gastric bubble and loops of bowel superior to the diaphragm, but plain radiography cannot reliably confirm the presence of the bowel within the pericardial sac. While prior traumatic cases have been diagnosed with echocardiography [6], this modality was noncontributory in our case, as our patient's heart could not be visualized, secondary to the massive herniation of stomach and bowel anterior to the heart obscuring the ultrasound beam. Our case demonstrates the utility of advanced multiplanar imaging modalities such as CMR in establishing the correct diagnosis.

## Conclusions

The true incidence of intrapericardial diaphragmatic hernias as a postoperative complication may have been underestimated prior to the advent of CMR. It is important to consider a diagnosis of intrapericardial diaphragmatic hernia in patients with pericardial windows who present with nonspecific cardiopulmonary or gastrointestinal symptoms. When such a diagnosis is suspect, and when conventional imaging modalities fail to yield a specific diagnosis, further evaluation with CMR should be considered.

## Consent

Written informed consent was obtained from the patient for publication of this case report and any accompanying images. A copy of the written consent is available for review by the Editor-in-Chief of this journal.

## Competing interests

The authors declare that they have no competing interests.

## Authors' contributions

BCD drafted the manuscript. HLC helped draft the manuscript. DWE acquired and interpreted the CMR images and helped draft the manuscript. All authors read and approved the final manuscript.

## Authors' information

BCD, MD, Resident of Internal Medicine. HLC, MD, Fellow of Cardiology. DWE, MD, Assistant Professor of Radiology and Internal Medicine Section on Cardiology.

## Supplementary Material

Additional file 1 **CMR Video 1**. Axial SSFP cine sequence through inferior portions of right and left ventricle demonstrates stomach (immediately anterior to apex) and portions of transverse colon within the pericardial sac. Also noted are bilateral pleural effusions and areas of consolidation within the right lung base. Click here for file

Additional file 2 **CMR Video 2**. Axial SSFP cine sequence at level of aortic and pulmonic valves demonstrates a long segment of the mid transverse colon anterior to pulmonic valve. Bilateral pleural effusions and consolidative changes in the right lung are also shown. Click here for file
