# Supplementary figures and images for: Iatrogenic intrapericardial diaphragmatic hernia diagnosed by cardiovascular magnetic resonance
Source: J Cardiovasc Magn Reson. 2010 Jan 8;12(1):3. doi: 10.1186/1532-429X-12-3 (PMC2817870; doi:10.1186/1532-429X-12-3)

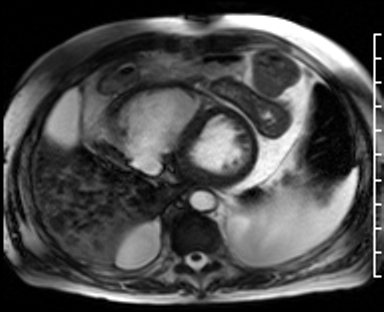

Supplement: Additional file 1 — CMR Video 1. Axial SSFP cine sequence through inferior portions of right and left ventricle demonstrates stomach (immediately anterior to apex) and portions of transverse colon within the pericardial sac. Also noted are bilateral pleural effusions and areas of consolidation within the right lung base. [file 1532-429X-12-3-S1.GIF]

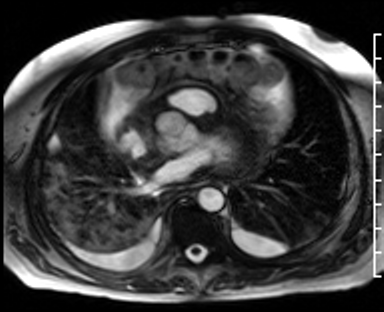

Supplement: Additional file 2 — CMR Video 2. Axial SSFP cine sequence at level of aortic and pulmonic valves demonstrates a long segment of the mid transverse colon anterior to pulmonic valve. Bilateral pleural effusions and consolidative changes in the right lung are also shown. [file 1532-429X-12-3-S2.GIF]
